# Audiometric Age-Related Hearing Loss and Cognition in the Hispanic Community Health Study

**DOI:** 10.1093/geroni/igab046.1327

**Published:** 2021-12-17

**Authors:** Justin Golub, Adam Brickman, Adam Ciarleglio, Nicole Schupf, José Luchsinger

**Affiliations:** 1 Columbia University, New York, New York, United States; 2 George Washington University, Washington, DC, District of Columbia, United States; 3 Columbia University, Mailman School of Public Health, New York, New York, United States

## Abstract

Studies associating age-related hearing loss (HL) with cognition have been limited by non-Hispanic cohorts, small samples, or limited confounding control. We overcome these limitations in the largest study of formal, audiometric HL and cognition to date using the multicentered Hispanic Community Health Study (n=5,277, mean age=58.4 [SD=6.2]). The main exposure was audiometric HL. The main outcome was neurocognitive performance. Adjusting for demographics, hearing aid use, and cardiovascular disease, a 20-dB increase (one-category worsening) in HL was cross-sectionally associated with worse performance in multiple neurocognitive measures: -1.53 (95% CI = -2.11, -0.94) raw score point difference on Digit Symbol Substitution Test, -0.86 (-1.23, -0.49) on Word Frequency Test, -0.76 (-1.04, -0.47) on Spanish-English Verbal Learning Test (SEVLT) 3 trials, -0.45 (-0.60, -0.29) on SELVT recall, -0.07 (-0.12, -0.02) on Six-Item Screener. Because HL is common and potentially treatable, it should be investigated as a modifiable risk factor for neurocognitive decline/dementia.

